# Amyloid fibril formation kinetics of low-pH denatured bovine PI3K-SH3 monitored by three different NMR techniques

**DOI:** 10.3389/fmolb.2023.1254721

**Published:** 2023-11-17

**Authors:** Luis Gardon, Nina Becker, Nick Rähse, Christoph Hölbling, Athina Apostolidis, Celina M. Schulz, Kevin Bochinsky, Lothar Gremer, Henrike Heise, Nils-Alexander Lakomek

**Affiliations:** ^1^ Institute of Biological Information Processing (IBI-7: Structural Biochemistry), JuStruct: Jülich Center for Structural Biology, Forschungszentrum Jülich, Jülich, Germany; ^2^ Institut für Physikalische Biologie, Heinrich-Heine-Universität Düsseldorf, Düsseldorf, Germany

**Keywords:** amyloid fibrils, fibrillation, aggregation kinetics, NMR spectroscopy, PI3K-SH3, neurodegeneration, atomic force microscopy

## Abstract

**Introduction:** Misfolding of amyloidogenic proteins is a molecular hallmark of neurodegenerative diseases in humans. A detailed understanding of the underlying molecular mechanisms is mandatory for developing innovative therapeutic approaches. The bovine PI3K-SH3 domain has been a model system for aggregation and fibril formation.

**Methods:** We monitored the fibril formation kinetics of low pH-denatured recombinantly expressed [U-^13^C, ^15^N] labeled bovine PI3K-SH3 by a combination of solution NMR, high-resolution magic angle spinning (HR-MAS) NMR and solid-state NMR spectra. Solution NMR offers the highest sensitivity and, therefore, allows for the recording of two-dimensional NMR spectra with residue-specific resolution for individual time points of the time series. However, it can only follow the decay of the aggregating monomeric species. In solution NMR, aggregation occurs under quiescent experimental conditions. Solid-state NMR has lower sensitivity and allows only for the recording of one-dimensional spectra during the time series. Conversely, solid-state NMR is the only technique to detect disappearing monomers and aggregated species in the same sample by alternatingly recoding scalar coupling and dipolar coupling (CP)-based spectra. HR-MAS NMR is used here as a hybrid method bridging solution and solid-state NMR. In solid-state NMR and HR-MAS NMR the sample is agitated due to magic angle spinning.

**Results:** Good agreement of the decay rate constants of monomeric SH3, measured by the three different NMR methods, is observed. Moderate MAS up to 8 kHz seems to influence the aggregation kinetics of seeded fibril formation only slightly. Therefore, under sufficient seeding (1% seeds used here), quiescent conditions (solution NMR), and agitated conditions deliver similar results, arguing against primary nucleation induced by MAS as a major contributor. Using solid-state NMR, we find that the amount of disappeared monomer corresponds approximately to the amount of aggregated species under the applied experimental conditions (250 µM PI3K-SH3, pH 2.5, 298 K, 1% seeds) and within the experimental error range. Data can be fitted by simple mono-exponential conversion kinetics, with lifetimes τ in the 14–38 h range. Atomic force microscopy confirms that fibrils substantially grew in length during the aggregation experiment. This argues for fibril elongation as the dominant growth mechanism in fibril mass (followed by the CP-based solid-state NMR signal).

**Conclusion:** We suggest a combined approach employing both solution NMR and solid-state NMR, back-to-back, on two aliquots of the same sample under seeding conditions as an additional approach to follow monomer depletion and growth of fibril mass simultaneously. Atomic force microscopy images confirm fibril elongation as a major contributor to the increase in fibril mass.

## Introduction

Protein misfolding, aggregation, and formation of amyloid fibrils are a hallmark of neurodegenerative diseases (Bucciantini et al., 2002; Dobson, 2003; Polverino de Laureto et al., 2003; Ross and Poirier, 2004; Chiti and Dobson, 2006; Chiti and Dobson, 2017; Iadanza et al., 2018; Willbold et al., 2021). A detailed understanding of the aggregation mechanism is mandatory to counteract the progression of neurodegenerative diseases and to develop appropriate medication. The Src-homology 3 domain from the p85α subunit of phosphatidyl-inositol-3-kinase (PI3K-SH3) is one of the best-investigated systems in terms of protein folding, aggregation, and fibrillation (Guijarro et al., 1998; Zurdo et al., 2001; Polverino de Laureto et al., 2003; Bader et al., 2006; Vettore and Buell, 2019). SH3 domains play a role in multiple signaling pathways by recognizing proline-rich PxxP motifs and mediating protein-protein interactions (Feng et al., 1994; Musacchio et al., 1994; Batra-Safferling et al., 2010; Saksela and Permi, 2012; Kurochkina and Guha, 2013; Dionne et al., 2022). Below pH 4, the PI3K-SH3 domain denatures, however, and forms fibrils (Guijarro et al., 1998; Zurdo et al., 2001). Solid-state NMR and cryo-electron microscopy have obtained detailed, high-resolution 3D-structural insights into bovine PI3K-SH3 fibrils (Bayro et al., 2010; Röder et al., 2019).

Several methods exist to measure the aggregation kinetics of amyloid formation. Amyloid dyes like Thioflavin T (ThT) or Congo red are frequently used as extrinsic probes to study the formation or presence of fibrils (Levine, 1993; Chiti et al., 1999; Biancalana and Koide, 2010; Schutz et al., 2011; Frieg et al., 2020). Also, methods employing intrinsic probes, such as tryptophan fluorescence or dynamic light scattering, can be used to follow the kinetics of amyloid formation or amyloid decomposition (Röder et al., 2019; Vettore and Buell, 2019; Housmans et al., 2021). NMR spectroscopy has been very powerful in elucidating the structures of amyloid fibrils (Balbach et al., 2000; Petkova et al., 2002; Jaroniec et al., 2004; Heise et al., 2005; Luhrs et al., 2005; Paravastu et al., 2008; Wasmer et al., 2008; Bayro et al., 2010; Schutz et al., 2011; Colvin et al., 2016; Tuttle et al., 2016; Wälti et al., 2016; Weirich et al., 2016; Gremer et al., 2017; Iadanza et al., 2018; Becker et al., 2023). But NMR can also follow protein fibrillation and aggregation kinetics using nuclear spins as an intrinsic probe. For example, Zurdo et al. studied the aggregation behavior of denatured PI3K-SH3 by employing one-dimensional solution NMR (Zurdo et al., 2001), combined with far-UV circular dichroism (CD) spectroscopy, Fourier-transform infrared (FTIR) spectroscopy and electron microscopy (EM). More recently, several studies investigated the fibril formation kinetics of Amyloid-β Aβ(1–40) and Aβ(1–42) and α-synuclein. Roche et al. and Bellomo et al. followed the fibril formation kinetics of Aβ(1–40) and Aβ(1–42) by a combination of solution NMR spectroscopy and ThT fluorescence (Roche et al., 2016; Bellomo et al., 2018), and Horvath et al. fibril elongation of α-synuclein (Horvath et al., 2023). In these studies, the disappearance of the monomer signal in solution was monitored. Fibrils or larger aggregates are difficult to detect by solution NMR due to the overall tumbling limitation, restricting the maximum weight of the molecular complex that is still visible to solution NMR. ThT fluorescence was chosen as a complementary method to detect the fibrils or oligomers originating from the aggregation process.

In contrast to solution NMR, solid-state NMR does not face any overall tumbling limitation, and high molecular weight systems can be studied. Fibrils or large aggregates become accessible via dipolar CP (cross-polarization) transfer. Also, mobile/flexible monomers can be detected via scalar-based transfers, such as, e.g., INEPT (Insensitive Nuclei Enhanced by Polarization Transfer). However, the absence of weight limitation in solid-state NMR comes at the cost of line broadening due to anisotropic interactions. To reduce the line broadening, magic angle spinning (MAS) is applied in solid-state NMR, which, to first order, averages the anisotropic interactions (anisotropic chemical shift and dipolar couplings). Ravera and coworkers investigated the fibril formation kinetics of Aβ(1–40) and Aβ(1–42) by solid-state NMR under MAS conditions (*in situ* SedNMR) (Bertini et al., 2013). The disappearance of the monomeric signal was monitored by one-dimensional ^1^H-^13^C INEPT spectra and the appearance of the aggregated species by one-dimensional ^1^H-^13^C CP spectra. Debelouchina and coworkers observed the maturation of liquid droplets of a low-complexity domain of FUS by MAS NMR-spectroscopy (Berkeley et al., 2021). One concern with studying fibril formation and aggregation kinetics by solid-state NMR spectroscopy has been magic angle spinning. MAS with frequencies of several kHz leads to centrifugal forces inside the rotor comparable to those experienced in an ultracentrifuge (Ravera, 2014; Wang et al., 2018). The aggregated protein in the sample is compacted at the rotor walls during spinning. The influence of agitated conditions induced by magic angle spinning on fibril formation and aggregation kinetics has not been thoroughly investigated.

High-resolution magic angle spinning (HR-MAS) solid-state NMR is a hybrid between solution NMR and solid-state NMR. In a simplified view, it can be considered as applying mainly solution NMR spectroscopy under slow magic angle spinning conditions to average sample inhomogeneities. HR-MAS has been used in the context of metabolomics or pharmaceutical research when the investigated samples are too dense or inhomogeneous to be studied by standard solution NMR (Lindon et al., 2009; Sitter et al., 2009; Holzgrabe, 2010). Only a few studies have applied HR-MAS in the context of aggregation processes (Warrass et al., 2000; Wang et al., 2018; Sahoo et al., 2020).

In the current study, we report the seeded fibril formation kinetics for the bovine PI3K-SH3 domain as an amyloid model protein using three different NMR methods: solution NMR, solid-state MAS NMR, and HR-MAS NMR spectroscopy. We used identical protein concentration, buffer conditions, and temperature for the aggregation measurements to allow maximal comparability between the different methods. We find that under the chosen conditions, notably including fibril seeds as nucleation points, magic angle spinning has only a weak influence on the aggregation kinetics, and we observe similar aggregation kinetics for solution NMR under quiescent conditions and HR-MAS and solid-state MAS experiments representing agitated conditions. Hence, solid-state MAS NMR seems suitable for following amyloid fibril formation and aggregation kinetics of proteins in general. In combination with solution NMR, residue-specific resolution of the decaying monomeric species can be obtained in a time series of two-dimensional solution NMR spectra.

## Materials and methods

### Protein expression and purification of bovine [U-^13^C, ^15^N] PI3K-SH3

Bovine PI3K-SH3 domain was expressed in *Escherichia coli* BL21 (DE3) as an N-terminal glutathione S-transferase (GST)-fusion followed by a thrombin protease cleavage site using pGEX-4T (GenScript Biotech, Netherlands) as vector plasmid. The 86 amino acid sequence of the purified bovine PI3K-SH3 domain used in this study is GS MSAEGYQYRA LYDYKKEREE DIDLHLGDIL TVNKGSLVAL GFSDGQEAKP EEIGWLNGYN ETTGERGDFP GTYVEYIGRK KISP with the N-terminal Gly-Ser residues remaining as overhang from the thrombin cleavage.

BL21 cells were grown in M9 medium (for [U-^13^C, ^15^N] labeling with 3.2 g U-^13^C-glucose and 2 g ^15^N-ammonium chloride per l culture medium as the sole carbon and nitrogen sources) at 37°C for ∼4.5 h until an OD_600_ of 0.6 was reached. Protein expression was induced by the addition of 0.5 mM Isopropyl β-d-1-thiogalactopyranoside (IPTG). The cells were then grown overnight at 28°C, harvested by centrifugation (5.000xg, 15 min) and resuspended in 5–8 mL per g cell mass of lysis buffer (50 mM 4-(2-hydroxyethyl)-1-piperazineethanesulfonic acid (HEPES) and 100 mM NaCl, 0.3 mM phenylmethylsulfonyl fluoride (PMSF) pH 7.6 containing 5 mg/mL lysozyme). After sonication with a Bandelin Sonopuls sonicator (Bandelin) using a VS 70T probe (60% amplitude, 3 × 5 min, 3 s “on,” 5 s “off,” ice cooling) and ultracentrifugation in an Optima XPN-80 ultracentrifuge (Beckman) equipped with a 70Ti rotor at 4°C and 42.000 rpm for 1 h, the supernatant was loaded on two serially connected 5 mL Protino GST/4B columns (Macherey-Nagel, Düren, Germany) equilibrated with 50 mM HEPES-NaOH buffer, 100 mM NaCl, pH 7.6, and 3 mM Dithiothreitol (DTT). The protein was eluted with 20 mM reduced glutathione in 50 mM HEPES, 100 mM NaCl, 3 mM DTT, and pH 7.6. The fractions containing the eluted GST-PI3K-SH3 fusion protein were cleaved using 5–7.5 NIH units of thrombin (SERVA Electrophoresis GmbH, Heidelberg, Germany) per mg protein for 2 days at 4°C under mild shaking conditions. Then, the solution was loaded on a Superdex 75 HiLoad 16/600 size exclusion column (GE Healthcare Europe GmbH, Freiburg, Germany), equilibrated, and run with 5 mM ammonium acetate (pH 7.7) as a volatile buffer system, leading to the separation of the cleaved GST from PI3K-SH3. To ensure complete removal of GST traces, fractions containing PI3K-SH3 were pooled and loaded again on two serially connected 5 mL Protino GST/4B columns (Macherey-Nagel, Düren, Germany) equilibrated with 5 mM ammonium acetate. The flow-through fractions containing purified bovine [U-^13^C, ^15^N] PI3K-SH3 were pooled, aliquoted, and freeze-dried for further usage. Analysis by SDS-PAGE and NMR suggest bovine PI3K-SH3 purities above 97%, as neither method detected impurities.

### Production of unlabeled PI3K-SH3 fibrils seeds

The production of bovine PI3K-SH3 fibril seeds was essentially carried out according to Röder et al. (2019). In brief, 100 nmol lyophilized non-isotopically labeled PI3K-SH3 was resuspended in 500 µL 10 mM glycine-hydrochloride pH 2.5 buffer, leading to a final concentration of 200 µM PI3K-SH3. The glycine-hydrochloride buffer comprised 10 mM glycine and 5.66 mM hydrochloric acid, resulting in a final pH 2.5. The solution was shaken in an Eppendorf tube at 1400 rpm at 42°C for 24 h. A ∼5 μL sample was withdrawn for Atomic Force Microscopy (AFM) measurements (see below). The remaining 495 μL were transferred into a 15 mL Falcon tube and then diluted to approximately 100 μM with another 500 μL 10 mM glycine-hydrochloride pH 2.5 buffer. The solution was then sonicated under ice-cooling for 15 s (1 s “on,” 2 s “off,” 10% amplitude) with a Bandelin Sonopuls sonifier using an M72 probe to disrupt the fibrils into smaller species called seeds.

### NMR sample preparation

100 nmol lyophilized bovine PI3K-SH3 was resuspended in 390 μL 10 mM glycine-hydrochloride buffer (pH 2.5, 8% D_2_O). The sample was divided into three parts (one for solution NMR, one for HR-MAS, and one for MAS solid-state NMR experiments). A total volume of 10 μL unlabeled PI3K-SH3 fibril seeds (100 μM stock) was added proportionally to each PI3K-SH3 sample, corresponding to a 1% (mol/mol monomer equivalents) seed mass. The final PI3K-SH3 protein concentration was 250 μM. The “dead time” (the time between the addition of seeds but before NMR spectra were recorded) was measured after adding the PI3K-SH3 fibril seeds.

### NMR spectroscopy

All NMR experiments described in the following were conducted at 298 K (25°C). The temperature at every spectrometer was calibrated using either Methanol (solution NMR probes, HR-MAS probe) or DSS (solid-state NMR probe). Details on all NMR experiments are listed in Supplementary Tables S1, S2.

#### Solution NMR measurements

A series of two-dimensional solution NMR ^1^H-^15^N HSQC spectra was recorded, with approximately 1 h duration each, to follow the disappearance of the monomeric species. Solution NMR measurements were conducted on a Bruker 900 MHz Avance Neo spectrometer equipped with a triple resonance ^1^H, ^13^C, ^15^N TCI cryoprobe, and for a second series of NMR experiments using a Bruker 600 MHz Avance III HD spectrometer equipped with a ^1^H, ^13^C, ^15^N triple resonance TCI cryoprobe.

#### HR-MAS experiments

A series of two-dimensional HR-MAS ^1^H-^15^N HSQC (^1^H detection) and one-dimensional ^1^H-^13^C INEPT (^13^C detection) spectra were recorded alternatingly; each experiment had a duration of approximately 1 h. HR-MAS NMR experiments were conducted on a Bruker Avance III HD 800 MHz spectrometer equipped with a 4 mm triple resonance ^1^H, ^13^C, ^15^N HR-MAS probe. The MAS frequency was set to 6 kHz.

#### Solid-state MAS experiments

A series of one-dimensional ^13^C-detected solid-state NMR ^1^H-^13^C CP experiments (duration: 1 h) and ^1^H-^13^C INEPT experiments (duration 1 h) were recorded alternatingly. The solid-state MAS experiments were conducted on a Bruker 600 MHz Avance NMR spectrometer equipped with a double resonance ^1^H, ^13^C 3.2 mm probe; the MAS frequency was set to 8 kHz. Field strengths for hard 90° pulses were 83 kHz for ^1^H and 45 kHz for ^13^C. For CP transfer, a field strength of 37 kHz for ^1^H and 45 kHz for ^13^C were used (Hartmann-Hahn zero quantum condition: $\omega_{13C}-\omega_{1H}=\omega_{MAS}$ ). The field strength for proton decoupling during acquisition was 82 kHz. The ^13^C-detected spectra were referenced indirectly using adamantane by setting its CH peak to 31.4 ppm (corresponding to the DSS reference scale).

#### Spectra analysis

All spectra were processed and integrated using the Bruker Topspin 4 software (Bruker, Billerica, MA, United States). Two-dimensional spectra were plotted using the CCPN Analysis 2.3 software (Vranken et al., 2005).

The first increment/FID for two-dimensional spectra was Fourier-transformed and integrated as a one-dimensional spectrum. The integration regions were 6.4–10.2 ppm for ^1^H-detected spectra and 5–75 ppm for the ^13^C-detected spectra. Error estimates were obtained by integrating a noise region in the 1D spectra. For CP spectra: 225 to 200 ppm (^13^C), for HSQC-based: 1.8 to −2 ppm (^1^H), for INEPT HR-MAS and solid state: −5 to −45 ppm (^13^C). For solid-state NMR spectra with ^13^C detection, the chemical shift dispersion is larger than for protons, limiting the (signal-free) noise regions. Therefore, for the ^13^C-detected CP and INEPT based spectra the integrated noise region differed, but was scaled to be proportional to the integrated signal region by multiplying it with the square root of the ratio (signal region width/noise region width).

The integrated intensities were fitted using the following equation:

$$I\left(t\right)=I_{0}*e^{-\left(\frac{t}{\tau }\right)}+const\left(\text{mono}-\text{exponential fit}\right)$$ (1)

For the INEPT experiments, *I(t)* corresponds to the monomer concentration in the solution. Besides the half-life time, *τ*, the initial signal intensity, *I*_*0*_, is a fitting parameter. Further, we have introduced a constant, *const*, considering that the monomer signal does not fully decay to zero (within the measurement time). This suggests a monomer/fibril equilibrium is reached, with a residual amount of monomer in the solution. For the CP-based experiments, *I(t)* corresponds to the monomer concentration that has become part of the fibril. The software Origin 19 (OriginLab Corporation, Northampton, MA, United States) was used for fitting the data.

Integration of the peaks in two-dimensional solution NMR spectra (Supplementary Figure S3) was performed using Topspin 4, integrating over a square of 0.1 ppm lateral length in the ^1^H and 1 ppm in the ^15^N dimension. The noise was integrated over a square of the same dimensions in an empty spectrum region.

### Atomic force microscopy (AFM)

After the NMR experimental series, all PI3K-SH3 samples were investigated using Atomic Force Microscopy (AFM). The aggregated solutions of HR-MAS and solid-state NMR rotors and the NMR tube were transferred to 2 mL Eppendorf tubes. For AFM samples of the fibril seeds, the solution was diluted in 10 mM glycine-hydrochloride, pH 2.5 buffer to a protein concentration of 0.5 μM; for HR-MAS and solid-state NMR AFM samples to 50 μM, and the liquid NMR sample to 5 μM. A 5–10 μL volume of the solutions was pipetted on a mica surface. After 10 min of incubation, the mica was washed extensively with Milli-Q water and dried under a nitrogen gas flush. The AFM micrographs were recorded in ScanAsyst mode using the peak-force tapping mechanism on a Bruker Multimode 8 (Billerica, Massachusetts, United States) using OMCL-AC160TS cantilevers (Shinjuku, Tokyo, Japan) at 1024 × 1024 pixel resolution. The images were processed with Gwyddion 2.61 (Nečas and Klapetek, 2012).

### Circular dichroism (CD) spectrometry

Far-UV-CD spectroscopy of bovine PI3K-SH3 samples was performed on a JASCO J-1500 CD spectropolarimeter (Jasco, Gross-Umstadt, Germany) using 1 mm path length quartz cuvettes (Hellma, Müllheim, Germany). Spectra were recorded at 20°C after dissolving lyophilized PI3K-SH3 to a protein concentration of 20 µM in 10 mM glycine-hydrochloride buffer, pH 2.5, or in 20 mM Na phosphate buffer, pH 6.8, with instrument settings as follows: step size 1 nm, scan speed 100 nm min^−1^, bandwidth 2 nm, DIT 4. Far-UV-CD spectroscopy data of bovine PI3K-SH3 fibrils were recorded at a protein concentration of 20 µM (monomer equivalents) in 10 mM glycine-hydrochloride buffer, pH 2.5, as described above. The signal-to-noise ratio was improved by accumulation of 6 scans per sample. The mean residue ellipticity, MRE or ${\left[\theta \right]}_{mrw}$ in deg·cm^2^·dmol^−1^ was calculated from the equation ${\left[\theta \right]}_{\text{mrw}}=\left(\theta_{\text{obs}}\times \text{MRW}\right)/\left(c\times d\times 10\right)$ , with $\theta_{\text{obs}}$ , observed ellipticity (in millidegrees); c , concentration (in g/l); d , cell path length (in cm); MRW (mean residue weight), molecular weight divided by number of peptide bonds.

### Thioflavin T (ThT) assay

For the fibril elongation assay, which was monitored through the amyloid-specific dye Thioflavin-T (ThT), fibril growth experiments were conducted in triplicates (using three separate batches). Each batch/sample contained 250 μM PI3K-SH3, 0.04% NaN_3_, and 1% seed material in 10 mM glycine-hydrochloride buffer, pH 2.5, using the same experimental conditions as the NMR measurements. The sample was incubated under quiescent conditions at 25°C for 80 h. Due to the limitations of ThT fluorescence measurements at low pH (Becker et al., 2023), every 8 h a 10 µL aliquot of the reaction mixture was withdrawn and diluted 1:10 in 20 mM sodium acetate buffer (pH 5.0), 50 mM NaCl, containing 20 μM ThT. Subsequently, the diluted samples were transferred to a 96-well half-area, non-binding surface, polystyrene plate (Corning, No. 3881, black with clear bottom), and the plate was sealed with clear sealing tape (232701, Thermo Scientific). The plate was then loaded into a FLUOstar Omega plate reader (BMG Labtech, Ortenberg, Germany). Fluorescence measurements were performed with an excitation wavelength of 448 nm and emission measurements at 482 nm at 25°C. Measurements were repeated every 20 s in cycles 1 to 500 to measure the sample for a total of 2.7 h for each data point of the original aggregation assay. For analysis, the plateau values of the ThT fluorescence curve between 2.2 and 2.7 h were averaged and plotted against the time.

## Results and discussion

### Solution NMR detects the decay of the monomeric species

**FIGURE 1:**
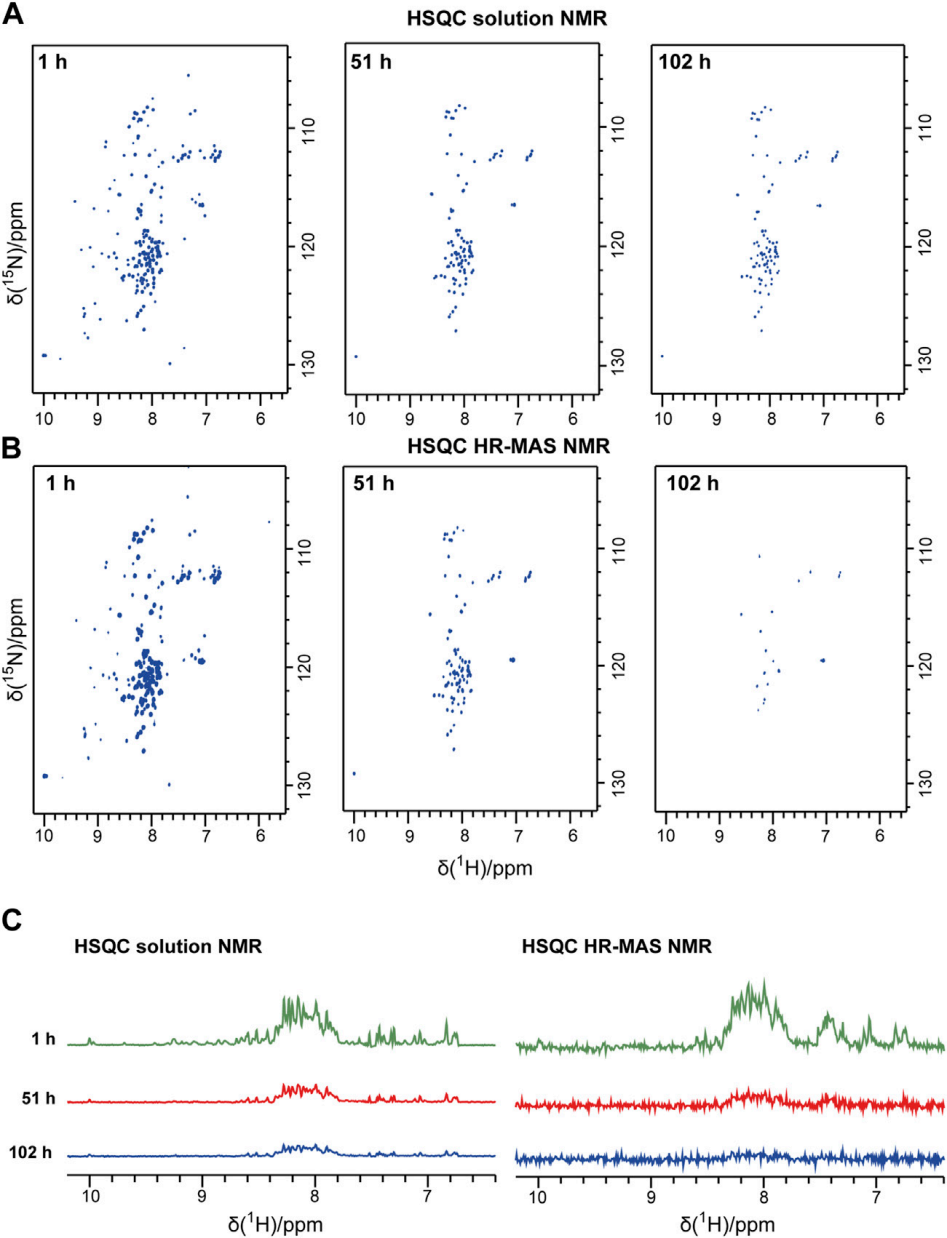
Solution NMR and HR-MAS NMR time series following the aggregation of the PI3K-SH3 monomers. Representative spectra recorded during the time series are shown: **(A)** Solution NMR ^1^H-^15^N HSQC spectra recorded under quiescent conditions. **(B)** HR-MAS ^1^H-^15^N NMR HSQC spectra were recorded at a MAS frequency of 6 kHz. In both ^1^H-^15^N spectra, the ^1^H resonance is correlated to the ^15^N resonance of the same amide group. Thus, one peak corresponds typically to one amino acid residue. **(C)** The first 1D increment of the 2D spectra is shown in **(A)** and **(B)**. All spectra were recorded at 25°C.

Bovine PI3K-SH3 loses its stable globular fold below pH 4 and aggregates into fibrils, as found previously by Dobson and coworkers (Zurdo et al., 2001). Here, we studied the PI3K-SH3 aggregation at low pH and under seeding conditions using three different NMR techniques (solution, HR-MAS, solid-state) and, for comparison, using a Thioflavin T assay. As a starting point, we dissolved lyophilized bovine PI3K-SH3 at pH 2.5 in 10 mM glycine-hydrochloride pH 2.5 buffer. We divided the sample into three aliquots of 250 µM final concentration (PI3K-SH3 monomer) for the three NMR experiments (solution, HR-MAS, solid-state) at a sample temperature of 25°C. To initiate immediate fibrillation, fibril seeds were added to each sample at a concentration of 1% molarity relative to the PI3K-SH3 monomer concentration in the solution. Initially, before fibrillation started, all PI3K-SH3 monomers were in solution and fully visible in the solution-state NMR spectra. To monitor the presence of the monomeric PI3K-SH3 species, we recorded a time series of consecutive two-dimensional (2D) solution NMR ^1^H-^15^N HSQC spectra of approximately one-hour duration each. Figure 1A shows 2D solution NMR ^1^H-^15^N HSQC spectra of unfolded monomeric bovine PI3K-SH3 at three exemplary time points after adding fibril seeds (1 h, 51 h, and 101 h). The narrow dispersion in the ^1^H dimension of the most intense peaks indicates that the major population of monomeric PI3K-SH3 is disordered under these conditions. However, a minor population of PI3K-SH3 with β-sheet conformation, similar to the globular conformation, is still present, visible as the less intense peaks with ^1^H chemical shifts ranging between 6 and 10 ppm. A direct comparison of ^1^H-^15^N HSQC spectra of PI3K-SH3 at pH 2.5 and PI3K-SH3 at pH 6.8 (globular conformation), see Supplementary Figure S1, underlines the presence of a minor folded species, similar to the globular protein at pH 6.8 and a major unfolded population. From the first increment of the solution NMR 2D spectra, shown in Figure 1C, we estimated the population of the minor folded species to approx. 15%–20%, based on intensities associated with the folded β-sheet region (9–10 ppm) compared to the central region between 7.5 and 8.5 ppm, which is dominated by intensities of the disordered conformation. Supplementary Figure S1 shows a direct comparison (overlay) of the 2D solution NMR spectra of SH3 at low and neutral pH. Supplementary Figure S2 shows the circular dichroism (CD) spectrum of PIK3-SH3 at pH 2.5, underlining that at pH 2.5 and 25°C, SH3 is mainly disordered. A CD spectrum of SH3 fibrils at the same pH of globular folded SH3 at neutral pH is compared. CD spectra agree with data shown in (Zurdo et al., 2001).

During the time course of aggregation, a series of solution NMR ^1^H-^15^N HSQC spectra were recorded. Spectra recorded at later time points show lower signal intensities of monomeric PI3K-SH3. Spectral intensities declined exponentially. The decay rate for the disordered and the minor folded conformation appears similar, as the inspection and comparison of the one-dimensional (1D) spectra of the first increments of the HSQC spectra indicates, see Figure 1C. Also, the intensities of representative peaks in the 2D solution NMR spectra decay with similar rate constants (Supplementary Figure S3). This suggests a dynamic equilibrium between the major unfolded population and the minor folded population, which must be slow on the NMR timescale (slower than about 10 ms, as two separate conformations are visible) but fast on the aggregation time scale (as all resonances decay with similar rate constants, in the order of hours by inspection of spectral intensities, see Figure 1 and Supplementary Figure S3). Intensities are decreasing because of increasing monomer aggregates during the experiment. The aggregated PI3K-SH3 species is not visible to solution NMR because of the high molecular weight of the aggregated species and the associated slow overall tumbling correlation time.

### Solid-state NMR detects the emerging aggregated species by CP-based methods and the decay of monomeric species by INEPT-based methods

**FIGURE 2:**
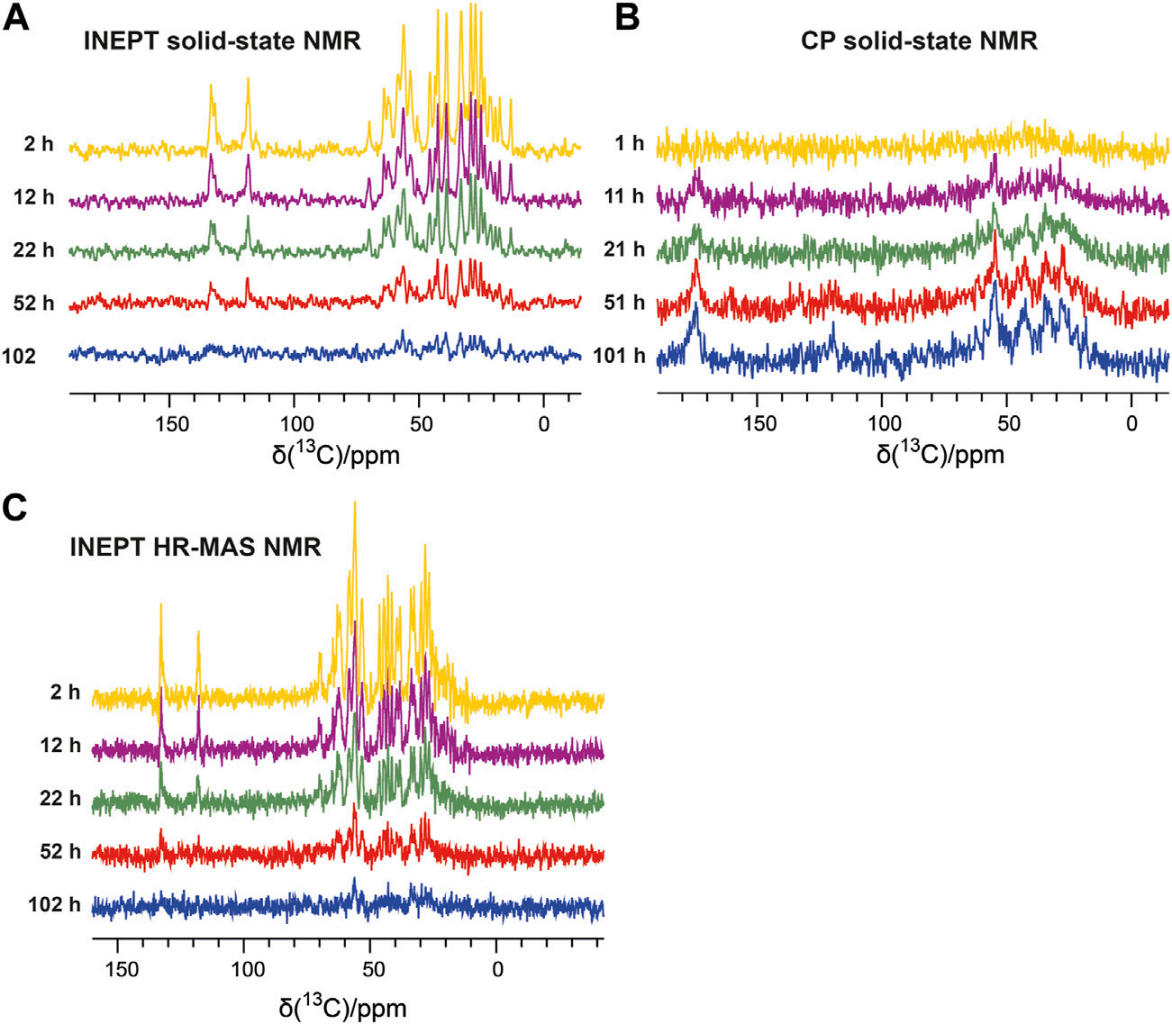
Solid-state NMR time series following the aggregation of the PI3K-SH3 monomers via the aliphatic (15–70 ppm) and aromatic carbon resonances (110–140 ppm). **(A)** ^13^C-detected ^1^H-^13^C INEPT solid-state spectra recorded at a MAS frequency of 8 kHz show the disappearance of the monomer. **(B)** One-dimensional ^13^C-detected ^1^H-^13^C cross-polarization (CP) MAS solid-state spectra recorded at a MAS frequency of 8 kHz detect the emergence of the aggregated species. **(C)** ^13^C-detected ^1^H-^13^C INEPT HR-MAS spectra were recorded at a MAS frequency of 6 kHz (without decoupling).

The emergence of the aggregated species can be followed by solid-state NMR, which does not face any overall weight limitation, as overall tumbling is absent in the solid state. Therefore, in parallel to the solution NMR time series, we recorded one-dimensional ^13^C-detected solid-state NMR spectra on the second aliquot of the P13K-SH3 sample (250 µM with 1% seeds added). Two types of magnetization transfer were used for the solid-state NMR spectra: In the first spectrum, a dipolar cross-polarization (CP) based transfer was employed, which is sensitive to the more rigid part of the sample, namely the aggregated species. Second, a scalar coupling-based INEPT (insensitive nuclei enhanced polarization transfer) was used, which is sensitive to the more flexible species, which is the unbound monomer in solution. Therefore, the INEPT-based 1D spectra connect to the solution-state NMR spectra on the monomeric species. Figures 2A, B show representative spectra of both time series, which were recorded inter-leaved. The CP-spectra (Figure 2B) show increasing intensities indicating the emergence of the aggregated species. This corresponds to a decay of the monomeric species, as visible in the INEPT-based spectra (Figure 2A).

To connect the observations by solution-state NMR and solid-state NMR, we also recorded a time series of interleaved ^1^H-^15^N HSQC spectra and ^13^C-detected INEPT spectra on the third aliquot of the sample, using an HR-MAS probe. This probe can record solution NMR-like spectra using proton-detection under MAS conditions, as in solid-state NMR. The influence of the magic angle spinning on the aggregation kinetics and the resulting product has not been investigated in detail yet. Figure 1B shows the time series of ^1^H-^15^N HSQC spectra recorded on bovine PI3K-SH3 under MAS conditions, with MAS frequencies of 6 kHz, recorded using an HR-MAS probe. Comparison of Figures 1A, B reveals that the initial PI3K-SH3 monomer spectra appear similar under both conditions, the quiescent condition during solution NMR experiments and under agitated conditions, namely centrifugal forces experienced during MAS conditions. However, the intensity decay rate appears slightly faster for the HR-MAS experiments than for the solution NMR experiments under quiescent conditions. This is also visible in the 1D spectra corresponding to the first increments of the respective HSQC spectra (Figure 1C) and will be discussed later. ^1^H-^15^N HSQC spectra, or their first increments, respectively, report on the protein backbone. A similar trend is observed for the HR-MAS ^13^C-detected INEPT spectra (Figure 2C), which are sensitive to protein sidechains. Consistent with the solid-state NMR spectra (Figure 2A), we observe that the concentration of the monomeric species decreases exponentially (see below) with time (Figure 2C).

**FIGURE 3:**
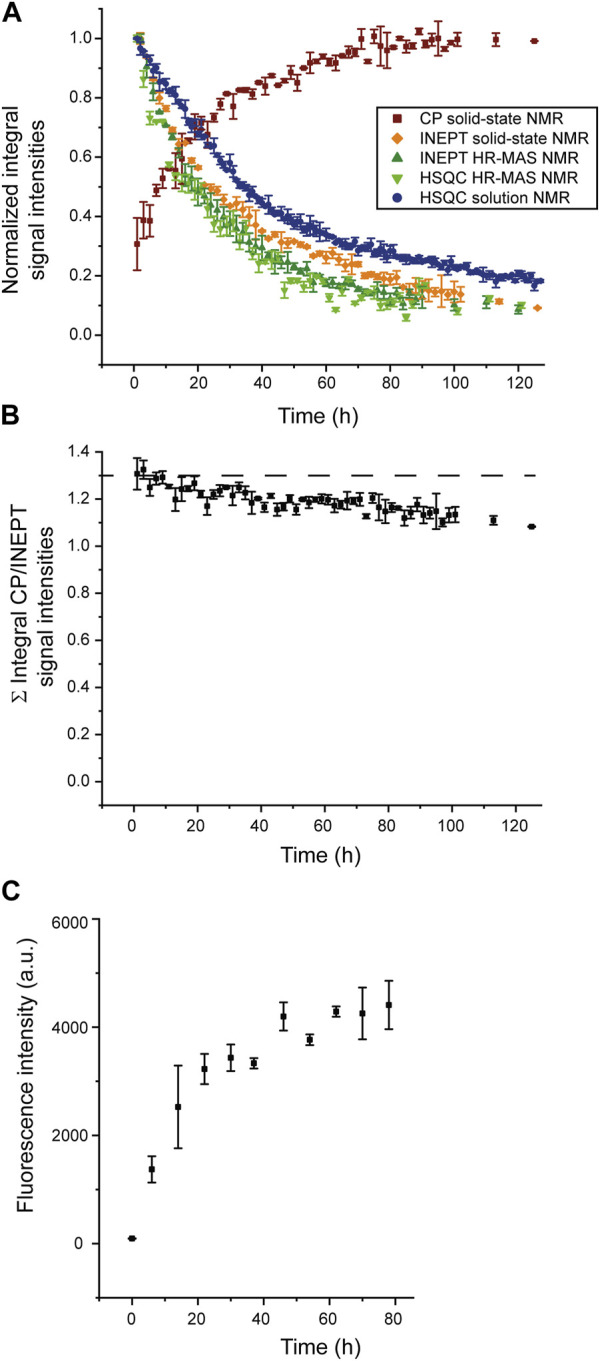
Normalized integrated intensities of NMR time series. **(A)** Normalized intensities of the spectra recorded at different time points during the time series, as shown in Figure 1 and Figure 2; CP-based 1D spectra (brown), first increments of solution NMR HSQC spectra (blue) or HR-MAS HSQC spectra (light green), 1D HR-MAS INEPT spectra (dark green) and INEPT solid-state NMR spectra (orange). (For the scalar coupling-based INEPT and HSQC experiments, sensitive to the monomeric species, intensities were normalized relative to the integrated intensity of the first 1D INEPT spectrum or first increment of the HSQC spectrum, respectively. For the CP-based 1D spectra, sensitive to the aggregated species, intensities were normalized relative to the last CP spectrum when spectral intensities have reached asymptotic behavior and do not grow any further. **(B)** Sum of normalized integrated intensities of the solid-state CP-based spectra, sensitive to the aggregated species, and the solid-state INEPT-based spectra, monitoring the decay of the monomeric species. **(C)** Increase of ThT fluorescence intensity due to binding to SH3 fibrils, monitored over time.

To analyze the aggregation kinetics extracted from the various NMR spectra more quantitatively, we integrated and normalized spectral intensities of the 1D spectra (or first increments of the 2D spectra), as described in the Materials and Methods section. The normalized integrated intensities as a function of time (after the start of the aggregation experiment) are shown in Figure 3A. Interestingly, in the case of the solid-state NMR CP- vs. INEPT-based 1D spectra, the sum of the two integrated intensities gives an almost constant value (Figure 3B). (We interpret the observed slight decrease of the sum of integrated intensities over several days as a slight detuning of the probe over time.) Thus, the aggregation appears as a two-state process, with a relatively direct transition from the monomeric to the fibrillar state, without a high population of an invisible oligomeric on-pathway intermediate. (Loss to an invisible off-pathway (“dead end”) oligomeric species that does not give rise to any CP-detected solid-state NMR signal, however, cannot entirely be excluded.) Mono-exponential decay curves (or the asymptotic growth curves, respectively) were fitted to the integrated intensities using Eq. 1 in the Materials and Methods section. The obtained half-life times, *τ*, are shown in Table 1. Supplementary Figure S4 gives details on the fits. Overall, the lifetimes measured using the different NMR techniques (solution-state, HR-MAS, solid-state) show good qualitative agreement. Under quiescent solution NMR conditions, the monomeric species seems to have a slightly longer lifetime than under MAS solid-state NMR conditions (37.4 ± 0.6 h vs. 30.2 ± 1.1 h), representing agitated conditions.

**TABLE 1:** Lifetime, *τ*, of the monomeric species (INEPT and HSQC) and build-up time, τ, of aggregated species (CP), as extracted from a mono-exponential fit of the normalized integrated intensities reported in Figure 3, see Supplementary Figure S4 for details on the fits.

Spectrum	*τ/h*	±Δ*τ/h*
CP solid-state	26.3	1.2
INEPT solid-state	30.2	1.1
INEPT HR-MAS (^13^C)	24.0	0.6
HSQC HR-MAS (^1^H)	25.0	1.2
HSQC solution NMR	37.4	0.6

For comparison, a ThT assay was recorded (Figure 3C), monitoring the increase of the fibrillar state. The observed increase in fibrillar structure is overall in good qualitative agreement with the solid-state NMR CP-based experiment, sensitive to the emergence of aggregated species (squared brown data points shown in Figure 3A). A comparison of the timeline of the CP-based solid-state NMR experiment and the ThT assay suggests that most CP signals originate from fibrillar SH3.

Integrated intensities of the first increment HR-MAS ^1^H-^15^N HSQC spectra, sensitive to the amide proton backbone signals of the monomer, decrease simultaneously to ^1^H-^13^C INEPT spectra, sensitive to the side chain signals, with almost identical lifetimes (25.0 ± 1.2 h vs. 24.0 ± 0.6 h). This suggests that both backbone or side-chain signals report unanimously on the decrease of the monomeric species. Under HR-MAS conditions, the decay of the monomeric species appeared slightly faster than under solid-state NMR conditions (*τ* = 24.0 ± 0.6 h vs. 30.2 ± 1.1 h). Thus, under both MAS conditions, the lifetime of the monomeric species appears slightly shorter than under quiescent solution NMR conditions (37.4 ± 0.6 h). We rationalize the slightly faster decrease of the monomeric species and, consequently, somewhat more rapid aggregation kinetics observed under agitated conditions in the MAS rotor by the influence of centrifugal forces on the aggregation process. Centrifugal forces will increase the local mass density close to the rotor wall. They may, therefore, slightly increase the likelihood of primary nucleation events, thus slightly speeding up the aggregation process.

To assess the reproducibility of the experimental setup, we repeated all experiments under identical conditions (Supplementary Figures S5, S6). While the results are in qualitative agreement, we observe differences when fitting the lifetimes of the monomeric species (from the INEPT spectra) and, in particular, for the build-up time of aggregated species (from the CP-based spectra), as described in Supplementary Table S3. Here, the observed differences in the lifetime of the monomeric species appear larger than the variation between the different NMR techniques (solution, solid-state, HR-MAS). Notably, in the second set of experiments, build-up times of aggregation as well as lifetimes of monomers in all experiments appear to be shortened to 50%–60% of the values in the original experiment, suggesting that this deviation is caused by an (unintended) variation of the starting conditions (e.g., slightly local differences in seed concentrations within the rotor or the NMR tube, respectively). This variation seems to have a larger influence than the NMR technique, making it difficult to judge or rationalize differences between the different NMR techniques fully. The way of seeding, or the seed itself, seems to have a major influence on the obtained aggregation kinetics. Also, we observe differences in the “appearance” time of the aggregated species extracted from the CP-based spectra. There will be a size distribution of aggregates during the aggregation process (see below). More stable and rigid aggregates like fibrils will contribute more strongly to the CP signal than smaller, more flexible species. Therefore, a slight variation of kinetics in the emergence of fibrils of different sizes will impact the growth of the CP signal over time.

### Atomic force microscopy images confirm fibril growths

**FIGURE 4:**
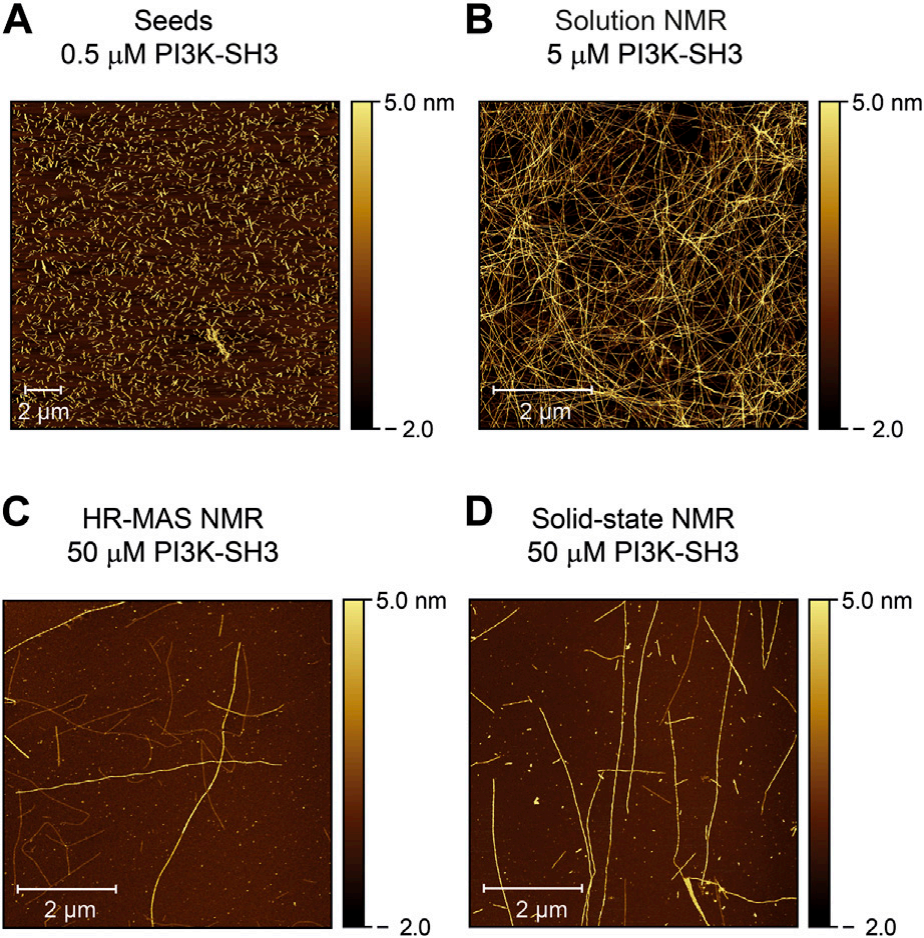
Atomic force microscopy images of PI3K-SH3 fibril seeds and the content of the NMR sample containers at the end of the fibrillation study were monitored by the three different NMR techniques (solution, HR-MAS, solid-state NMR). **(A)** AFM image of fibril seeds of bovine PI3K-SH3 used in this study. Fibril seeds produced by ultrasonication of PI3K-SH3 fibrils appear as short fibril fragments with approximate main lengths of ∼ 0.3–0.7 µm. Seeds were added at 1% molar monomer equivalent concentration to monomeric PI3K-SH3 for all aggregation experiments. **(B–D)** AFM micrographs of the PI3K-SH3 fibrils obtained at the end of the solution NMR **(B)**, HR-MAS **(C)**, and solid-state NMR **(D)** measurements. For AFM measurements, samples were diluted 1:50 for the solution NMR sample and 1:5 for the solid-state NMR and HR-MAS samples performed under MAS conditions. As visible from the AFM images, PI3K-SH3 fibril seeds substantially elongated to fibrils with several µm in length.

To check the morphology of the bovine PI3K-SH3 fibril seeds before and after the NMR time series, we recorded atomic force microscopy (AFM) images: First of the fibril seeds (Figure 4A) and second of all NMR samples, once the recording of the fibrillation time series was finished. Samples were taken from all sample containers of the three different NMR methods employed: solution NMR (Figure 4B), HR-MAS (Figure 4C), and solid-state NMR (Figure 4D). The consistency of all samples was solution-like and not gel-like. The AFM images show that elongated amyloid fibrils with several µm length were formed during all measurements, substantially longer than the sub-µm short fibrils used for seeding (Figure 4A). For the sample obtained from the quiescent solution NMR conditions (Figure 4B), a wealth of fibrils is visible in the AFM images. The observed fibril density is less for the HR-MAS (Figure 4C) and the solid-state NMR sample (Figure 4D), both recorded under MAS conditions. At the same time, the fibrils in the solution NMR sample show a homogenous distribution; fibrils in the samples obtained after the MAS experiments are distributed more inhomogeneous. However, the sample was obtained from the center of the rotor. At the same time, the highest local density of fibrils will be found close to the rotor wall due to the centrifugal forces present during the MAS sample rotation. In addition to separated PI3K-SH3 fibrils, a few large aggregates with unresolvable structures can be observed in the AFM images of the experiments involving a MAS rotor and sample rotation (solid-state NMR and HR-MAS). The faster aggregation kinetics observed in the second NMR time series results in shorter but also more fibrils (Supplementary Figure S7).

The observance of substantially elongated fibrils suggests that fibril elongation is the dominant mechanism for fibril formation. This is also in line with the observed exponential decay of the monomer concentration, considering that the monomer concentration decreases upon fibril formation and the monomer supply is not unlimited. Following Occam’s razor, we fitted the observed decay on the monomer concentration using a mono-exponential decay function (Supplementary Figure S4). If the decay rate of the monomer concentration, $\frac{d}{dt}c\left(monomer\right)$ , solely depends on the available monomer concentration (and the decay rate constant α), a simple differential equation can describe this $\frac{d}{dt}c\left(monomer\right)=-\alpha \;c\left(monomer\right)$ . The solution of that differential equation is a mono-exponential decay function $c\left(monomer\right)=c\left(0\right)*\exp\left(-\alpha *t\right)$ , with the initial monomer concentration *c*(0) and the decay constant $\alpha =\frac{1}{\tau }$ where τ is the fitted half-life time. This is consistent with the fits described in Supplementary Figure S4, where the fitted intensity corresponds to the monomer concentration (see also Materials and Methods).

## Conclusion

We have followed the seeded aggregation kinetics of denatured bovine PI3K-SH3, a model system for studying aggregation and amyloid fibril formation (Guijarro et al., 1998; Zurdo et al., 2001; Polverino de Laureto et al., 2003; Chiti and Dobson, 2006; Bayro et al., 2010; Chiti and Dobson, 2017; Röder et al., 2019), by a combination of three different NMR techniques: solution NMR, solid-state NMR, and HR-MAS NMR, providing the link between solution NMR and solid-state NMR. Scalar coupling-based INEPT experiments, which have been applied in all three NMR techniques, are sensitive to the disappearance of the monomeric species. In contrast, cross-polarization (CP) based solid-state NMR experiments detect the emergence of the aggregated species. Overall, we find good agreement between all three NMR techniques. The solid-state NMR experiments have the advantage of monitoring monomeric species’ disappearance and aggregated species’ emergence on the same sample (inside the solid-state NMR rotor). Here, the MAS spinning (representing agitated conditions) seems to accelerate the fibril formation kinetics only slightly compared to the quiescent solution NMR conditions. However, AFM data from the reaction under quiescent solution NMR conditions show the most homogenous fibril distribution. By solid-state NMR, under the investigated conditions, we find that the amount of disappeared monomer (visible by INEPT) corresponds to the increasing amount of the aggregated species (visible by CP), suggesting a two-state model for PI3K-SH3 aggregation and the absence of an oligomeric on-pathway intermediate, under the used conditions. Following Occam’s razor, the observed monomer-to-fibril kinetics, under seeding conditions, can be fitted using simple mono-exponential functions, both for the decay of PI3K-SH3 monomers as well as for the PI3K-SH3 fibrils’ build-up. AFM images, taken before and after the measurements, show that the initial fibril seeds substantially elongated during the NMR time series, suggesting fibril elongation as the dominant fibril growth mechanism.

For α-synuclein at neutral pH, fibril elongation has also been shown to be the dominant fibrillation mechanism (Buell et al., 2014b). However, below pH 6, secondary nucleation becomes the dominant mechanism for fibril generation. Here, one must distinguish between fibril growth and nucleation (Michaels et al., 2018). Fibril elongation is frequently the primary mechanism of growth in fibril mass (Meisl et al., 2017), while primary and secondary nucleation lead to the emergence of new fibrils (Meisl et al., 2017; Michaels et al., 2018). In the absence of seeds, primary nucleation is the only option for nucleation, whereas with increasing seed concentration secondary nucleation will become more important, as in the case of Aβ (Meisl et al., 2014). Indeed, a comprehensive set of parameters (monomer concentration, seed concentration, pH, temperature, quiescent vs agitated, etc.) will determine the balance between different fibrillation mechanisms (Buell et al., 2014a; Buell et al., 2014b; Michaels et al., 2018). Knowles and co-workers have derived a sophisticated Master equation approach to delineate the contribution of the various mechanisms (Knowles et al., 2009; Cohen et al., 2011b; Cohen et al., 2011c). Application of this approach requires a large set of experimental data, including variations in monomer concentration and seed concentration (Buell et al., 2014b; Meisl et al., 2017). The contributions of different nucleation and elongation mechanisms to the total fibrillation process can be analyzed by measuring the reaction half-time as a function of monomer concentration (Meisl et al., 2017). Nevertheless, fibril elongation is frequently the primary process of generating new fibril mass (Meisl et al., 2017).

As the intensity of the CP-based solid-state NMR spectra is proportional to the amount of fibril mass, and AFM data support the fibril growth by fibril elongation in the case of PI3K-SH3 (Figure 4), we argue for fibril elongation as the dominant mechanism of the observed increase of the CP-based solid-state NMR signal (Figure 3A). Indeed, in the presence of a large number of seeds and when fibril elongation is the dominant growth mechanism for generating new fibril mass, the aggregation profile is expected to be a single exponential function (Cohen et al., 2011a; Buell et al., 2014b). Secondary nucleation as an alternative mechanism, however, cannot be excluded. It may have occurred as a secondary mechanism responsible for the faster aggregation kinetics observed in the “reproduced” experiment (see above), where shorter and more fibrils were observed after fibrillation. However, faster monomer decay rates at higher temperatures (data not shown) argue against secondary nucleation as the dominant fibrillation mechanism, see, e.g. (Törnquist et al., 2018).

Our data support that solid-state NMR, in combination with AFM, presents a valuable addition to measure the kinetics of fibril mass increase. By comparing scalar coupling-based and CP-based solid-state NMR intensities (agitated conditions, by MAS rotation) with quiescent condition (solution NMR), we find that under sufficiently high seed concentrations (1% seeds in our case), primary nucleation events induced by MAS rotation, have only a small influence. By comparing solution NMR measurements, sensitive to the depletion of monomer, and solid-state NMR sensitive to fibril mass increase by CP-based measurements (but also to monomer depletion by conducting scalar coupling-based experiments as an internal reference), we followed fibril mass increase and monomer depletion simultaneously.

## Data Availability

The original contributions presented in the study are included in the article/Supplementary Material, further inquiries can be directed to the corresponding authors.
