# Supplementary material for: Amyloid fibril formation kinetics of low-pH denatured bovine PI3K-SH3 monitored by three different NMR techniques
Source: Front Mol Biosci. 2023 Nov 17;10:1254721. doi: 10.3389/fmolb.2023.1254721 (PMC10691488; doi:10.3389/fmolb.2023.1254721)
Supplement: Supplementary file 1 [file Table1.DOCX]

**Contribution to the field statement**

Amyloid fibril formation is a molecular hallmark of neurodegenerative diseases. Thioflavin T or Congo red assays are frequently applied to follow the fibril formation kinetics. Besides that, a few solution NMR studies monitored the disappearance of the monomer signal to follow the aggregation kinetics. In addition to solution NMR, Magic Angle Spinning (MAS) solid-state NMR can be employed, which has the advantage that both the decay of the monomer and the growth of the fibril can be studied with the same sample. However, only a few solid-state NMR studies have been conducted in that context.

We have followed the seeded aggregation kinetics of low-pH denatured bovine PI3K-SH3, a model system for studying aggregation and fibril formation by combining solution NMR, solid-state NMR, and high-resolution MAS NMR. We find good agreement between all three NMR techniques and only a small impact of magic angle spinning. NMR, in combination with atomic force microscopy (AFM) data, indicates that the amount of disappeared monomer corresponds to increasing fibrils, suggesting a two-state model for PI3K-SH3 fibrillation. The observed seeded monomer-to-fibril kinetics can be fitted using a simple mono-exponential decay function, suggesting fibril elongation as the dominant growth mechanism.
