# Supplementary material for: Amyloid fibril formation kinetics of low-pH denatured bovine PI3K-SH3 monitored by three different NMR techniques
Source: Front Mol Biosci. 2023 Nov 17;10:1254721. doi: 10.3389/fmolb.2023.1254721 (PMC10691488; doi:10.3389/fmolb.2023.1254721)
Supplement: Supplementary file 2 [file DataSheet1.pdf]

Supplementary Material

Amyloid fibril formation kinetics of low-pH denatured bovine PI3K-SH3 monitored by three different NMR techniques

Luis Gardon[1,2#], Nina Becker[1,2#], Nick Rähse[2], Christoph Hölbling[2], Athina Apostolidis[2], Celina M. Schulz[2], Kevin Bochinsky[1], Lothar Gremer[1,2*], Henrike Heise[1,2*], Nils-Alexander Lakomek[1,2*]

[1] Institute of Biological Information Processing (IBI-7: Structural Biochemistry) and JuStruct: Jülich Center for Structural Biology, Forschungszentrum Jülich, Jülich, Germany;

[2] Institut für Physikalische Biologie, Heinrich-Heine-Universität Düsseldorf, Düsseldorf, Germany.

[#] authors contributed equally and share the first authorship

[*] correspondence: l.gremer@fz-juelich.de, h.heise@fz-juelich.de, n.lakomek@fz-juelich.de

Table of contents:

1. Supplementary Materials and Methods:

Supplementary Table S1: Experimental parameters of 2D ^{1}H-^{15}N HSQC experiments (solution NMR and HR-MAS)

Supplementary Table S2: Experimental parameters of 1D ^{13}C-detected experiments

2. Supplementary Results:

Supplementary Figure S1: Comparison of solution NMR ^{1}H-^{15}N HSQC spectra of bovine PI3K-SH3 at neutral and acidic pH conditions.

Supplementary Figure S2: Far-UV-CD spectra of bovine PI3K-SH3 at pH 6.8 and 2.5

Supplementary Figure S3: Decay of representative intensities in two-dimensional solution NMR spectra.

Supplementary Figure S4: Fit of integrated intensities shown in Figure 3 and Figure S6

Supplementary Figure S5: Comparison of the integrated intensities of our first NMR measurements and our repeated (reproduced) NMR measurements.

Supplementary Figure S6: Fit of integrated intensities shown in Figure 3 and Figure S4.

Supplementary Table S3: Fitting parameters of decay and build-up curves (mono-exponential fit).

Supplementary Figure S7: AFM images of the repeated (reproduced) measurement.

1. Supplementary Materials and Methods

Supplementary Table S1: Experimental parameters of 2D ^{1}H-^{15}N HSQC experiments (solution NMR and HR-MAS).

	HSQC (solution NMR)	HSQC (solution NMR)	HSQC (HR-MAS)
Field (T)	21.15	14.1	18.8
^{1}H Larmor frequency	900 MHz	600 MHz	800 MHz
Sample volume	200 µl (3 mm tube)	200 µl (3 mm tube)	~110 µl (4 mm rotor)
MAS frequency (kHz)	-	-	6
Bruker pulse program	hsqcfpf3gpphwg	fhsqcf3gphh	hsqcfpf3ggphwg
Interscan recovery delay (s)	1.3	1.2	1
Number of scans	8	8	16
Dummy scans	4	16	4
Total experimental time	56 min 36 sec	56 min 39 sec	59 min 42 sec
**Direct dimension (^{1}H)**			
^{1}H carrier (ppm)	4.7	4.698	4.699
Spectral width (ppm)	16.3427	16.0205	16.0294
Data points	2048	1024	2048
Acquisition time (ms)	69.6	53.2	79.9
**Indirect dimension (^{15}N)**			
^{15}N carrier (ppm)	117	99.877	117
Spectral width (ppm)	32	79.9	35
Data points	300	328	200
Acquisition time (ms)	51.4	33.7	35.2

Supplementary Table S2: Experimental parameters of 1D ^{13}C-detected experiments.

	INEPT (HR-MAS)	INEPT (solid-state)	CP (solid-state)
Field (T)	18.8	14.1	14.1
^{1}H Larmor frequency	800	600	600
Sample volume	~110 µl (4 mm rotor)	~32 µl (3.2 mm rotor)	~32 µl (3.2 mm rotor)
MAS frequency (kHz)	6	8	8
Interscan recovery delay (s)	1.5	2	2
Number of scans	2368	1780	1780
Dummy scans	0	2	2
Total experimental time	1h 0 min 34 sec	1h 0 min 5sec	1h 0 min 1 sec
Transfer	^{1}H-^{13}C INEPT	^{1}H-^{13}C INEPT	^{1}H-^{13}C CP
^{13}C Carrier (ppm)	56	70	100
Duration of transfer (ms)	3.4	3.4	0.7
Spectral width (ppm)	202.7	251	251
Data points	2048	1024	1024
Acquisition time (ms)	25.1	13.5	13.5
Decoupling power during acquisition (kHz)	no decoupling	82	82

2. Supplementary Results

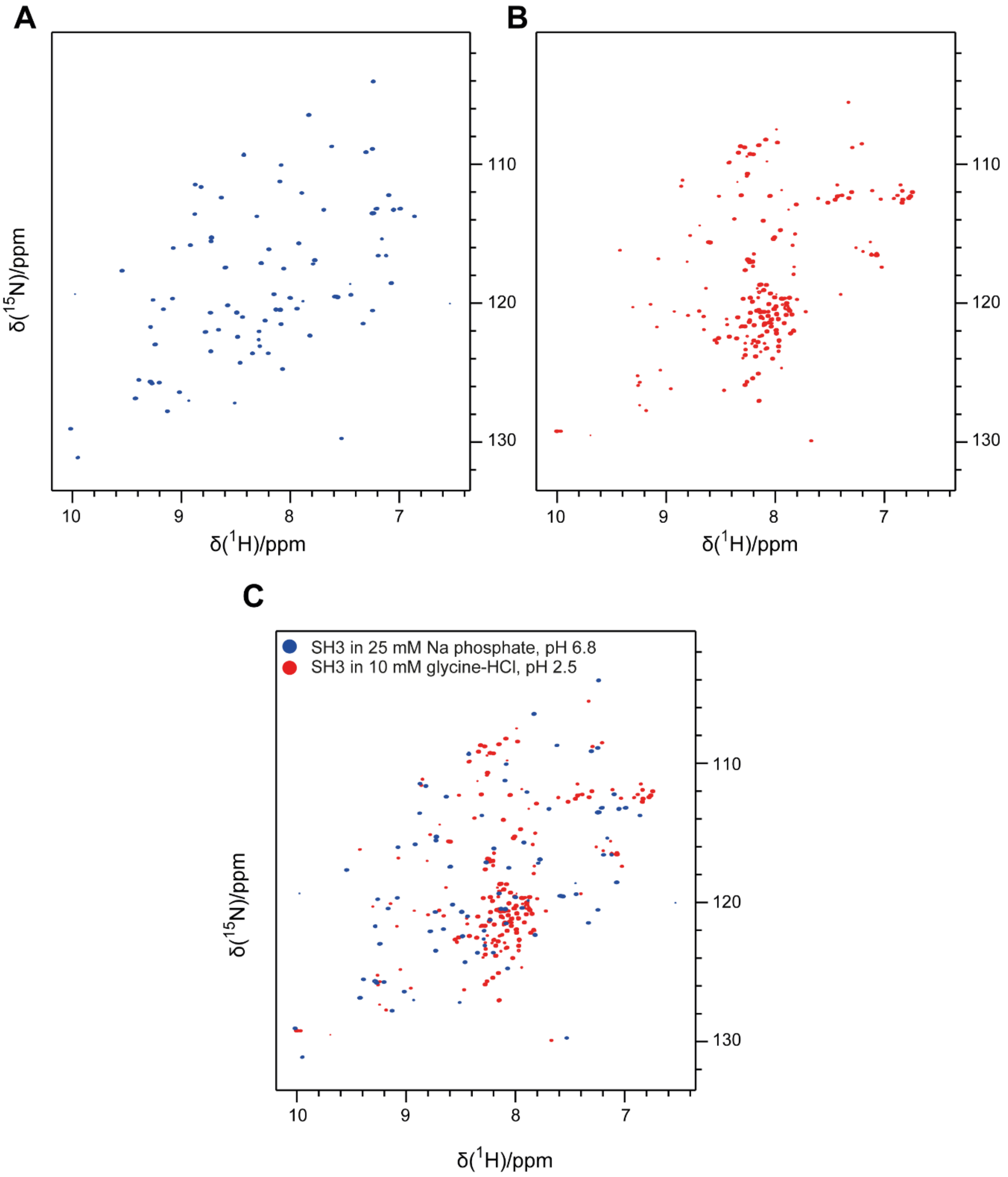

Supplementary Figure S1: Comparison of solution NMR ^{1}H-^{15}N HSQC spectra of bovine PI3K-SH3 at neutral (A, blue) and acidic (B, red) pH conditions. A) The blue spectrum was recorded on 250 µM $^{15}N^{13}C$ PI3K-SH3 sample dissolved in 25 mM sodium phosphate buffer at pH 6.8 and 900 MHz and 25 °C. It shows a wide signal dispersion expected for a β-strand-native structure of the PI3K-SH3 domain. B) The red spectrum was recorded on 250 µM $^{15}N^{13}C$ PI3K-SH3 sample dissolved in 10 mM glycine-HCl buffer at pH 2.5 and 900 MHz and 25 °C. A collapse of peaks into the region of 7.5 and 8.5 ppm, indicating disordered conformation, is observed in the red spectrum. However, peaks still appear in the region above 8.5 ppm, indicating β-strand content. C) Overlay of the solution NMR ^{1}H-^{15}N HSQC spectra of PI3K-SH3 at acidic (red) and neutral (blue) pH conditions shown in panels A and B).

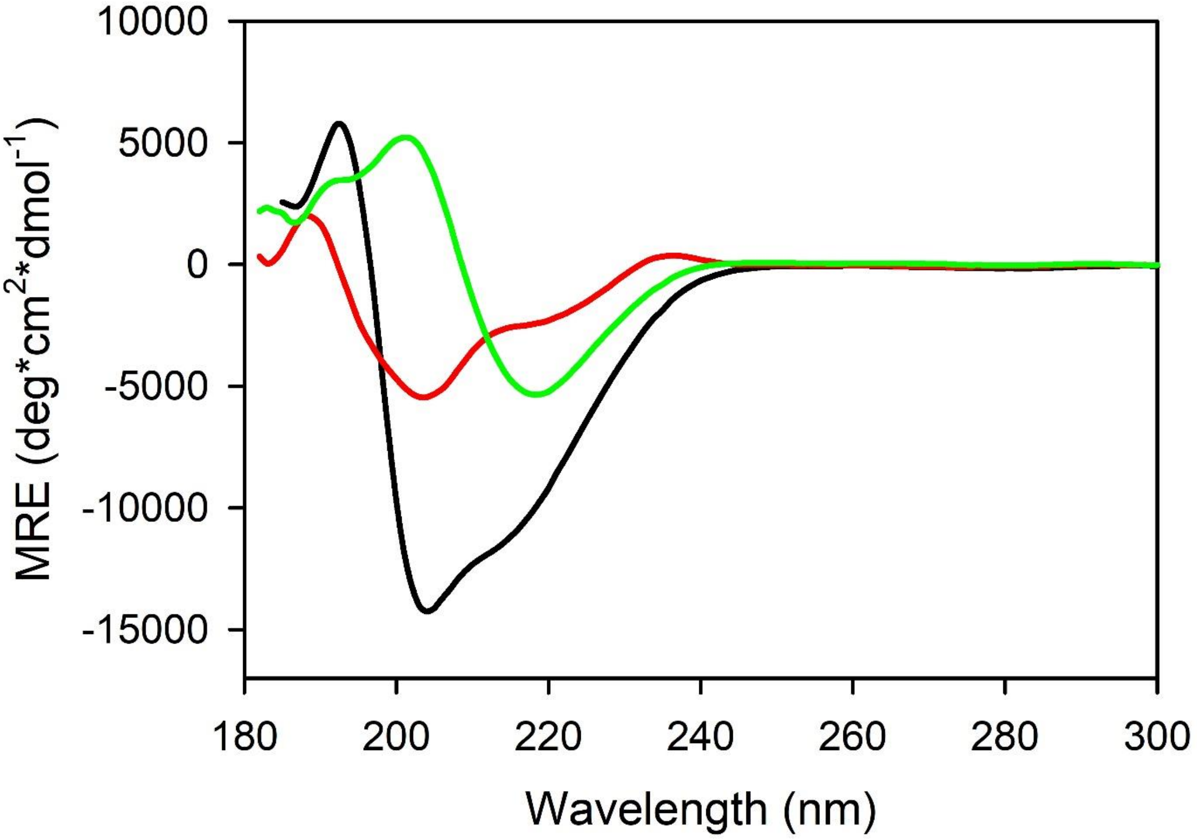

Supplementary Figure S2. Far-UV-CD spectra of bovine PI3K-SH3 in its monomeric state at pH 6.8 (red) or 2.5 (black) or in its fibrillar state at pH 2.5 (green), respectively. Far-UV-CD spectra were recorded at 20 µM protein concentration after dissolving lyophilized bovine PI3K-SH3 in 10 mM glycine-hydrochloride buffer, pH 2.5, or in 20 mM Na phosphate buffer, pH 6.8, respectively. Fibrils of bovine PI3K-SH3 were grown at 200 µM protein concentration overnight at 42°C in 10 mM glycine-hydrochloride buffer, pH 2.5, and diluted to 20 µM concentration (monomer equivalents) for CD measurements. MRE, mean residue ellipticity.

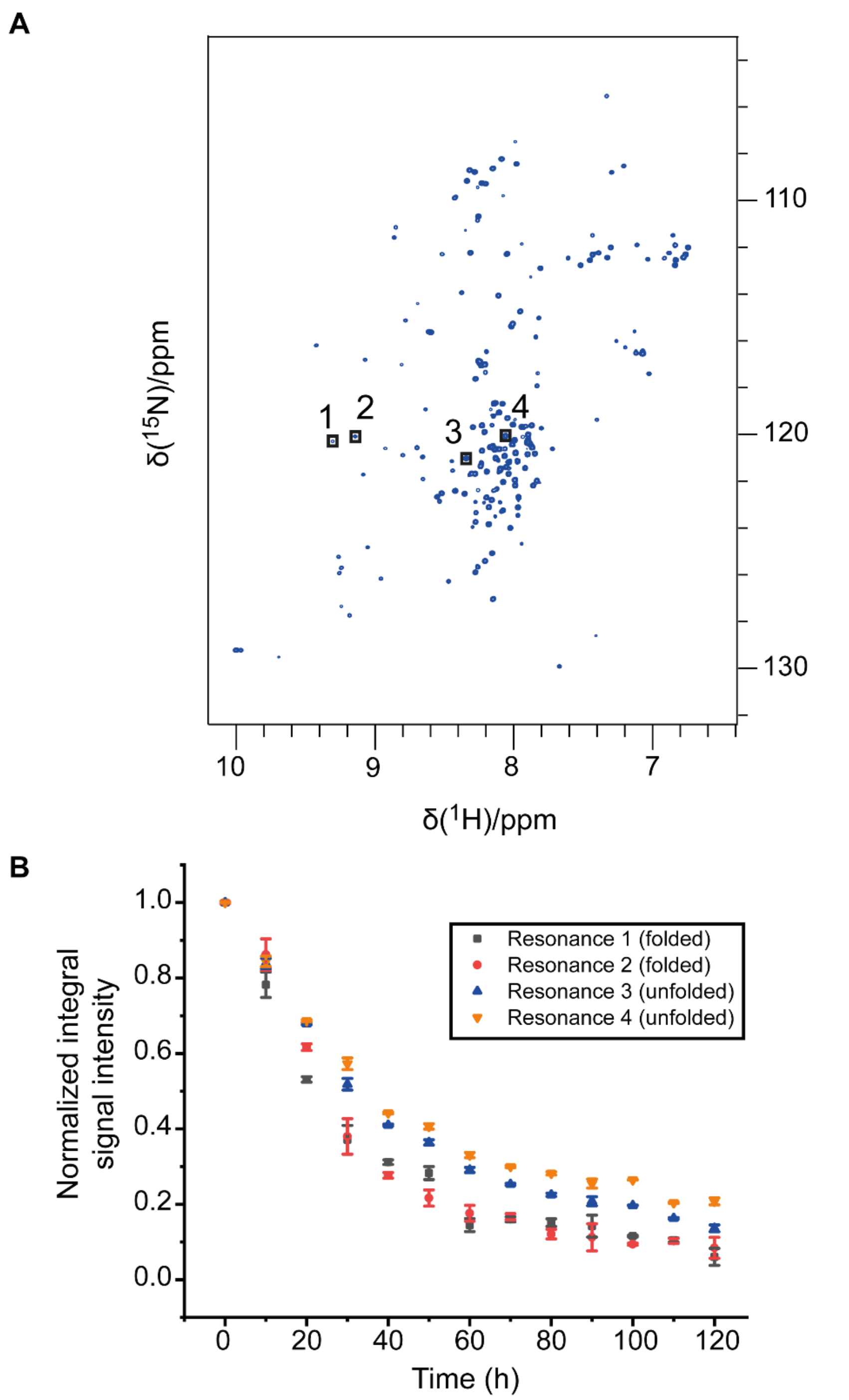

Supplementary Figure S3. Decay of representative intensities in two-dimensional solution NMR spectra. (A) Four representative peaks (NMR resonances) were selected from the two-dimensional solution NMR spectrum of SH3 at pH 2.5 and 25°C. Peaks (1) and (2) show ^{1}H chemical shifts that are characteristic of folded secondary structure elements, most likely β-sheet conformation. Peaks belonging to the unfolded region have a ^{1}H chemical shift range between 7.5 and 8.5 ppm. Most resonances in this region belong to the unfolded SH3 population. However, the resonances of the folded SH3 (minor) population overlap with this region. Peaks (3) and (4) presumably belong to the (minor) folded population. (B) Normalized intensity decay of the representative peaks (1-4) shown in (A).

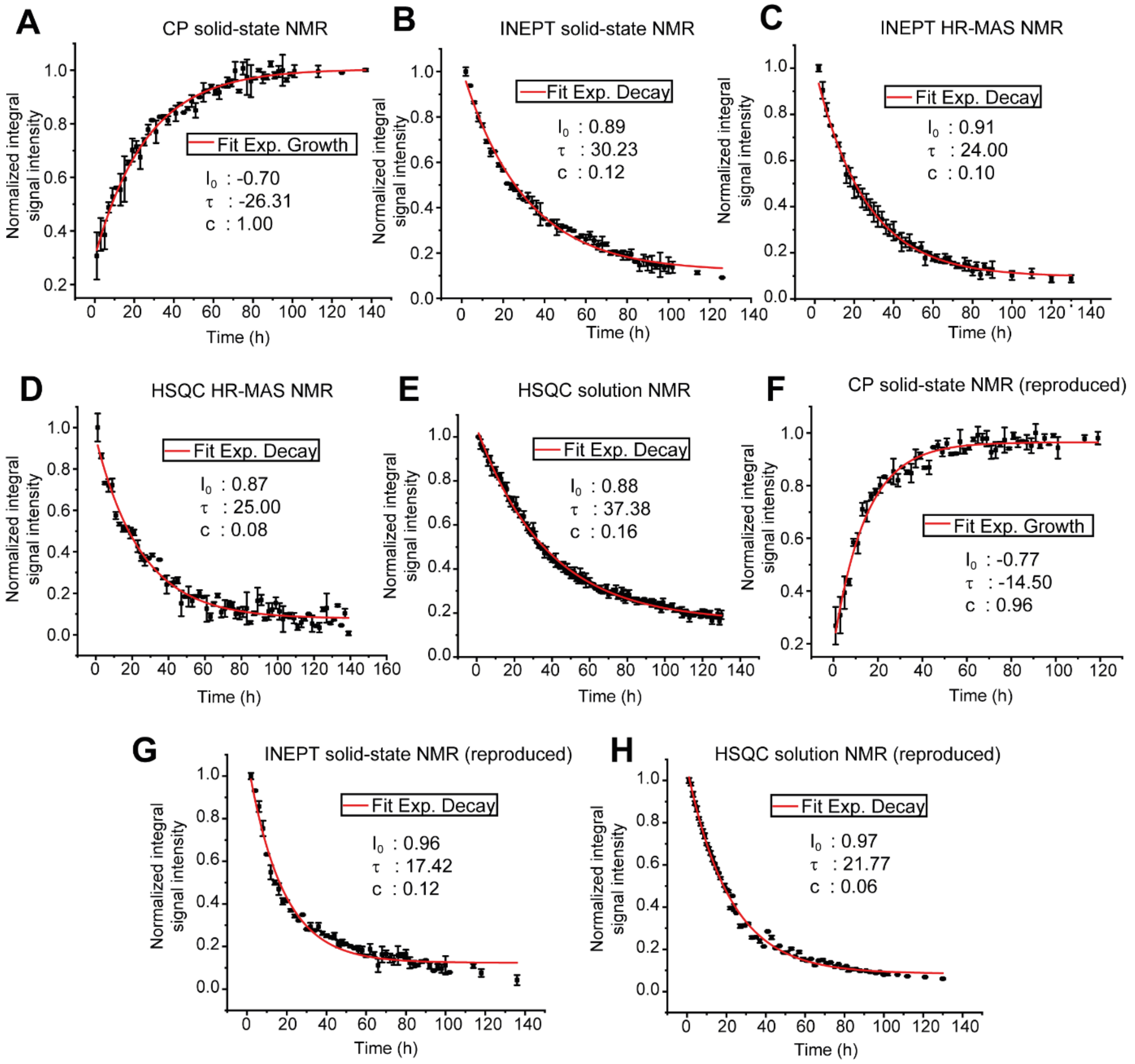

Supplementary Figure S4: Fit of integrated intensities shown in Figure 3 and Figure S6. Integrated intensities were fitted using the mono-exponential equation $I(t) = I_0 * e^{-\left(\frac{t}{\tau}\right)} + c$, with fitted parameters τ and c. A) CP-based solid-state NMR spectrum, B) INEPT-based solid-NMR spectrum, C) INEPT-based HR-MAS NMR spectrum, D) HSQC-based HR-MAS spectrum, E) HSQC-based solution NMR spectrum, F) CP-based solid-state NMR spectrum (reproduced), G) INEPT-based solid-NMR spectrum (reproduced), H) HSQC-based solution spectrum (reproduced).

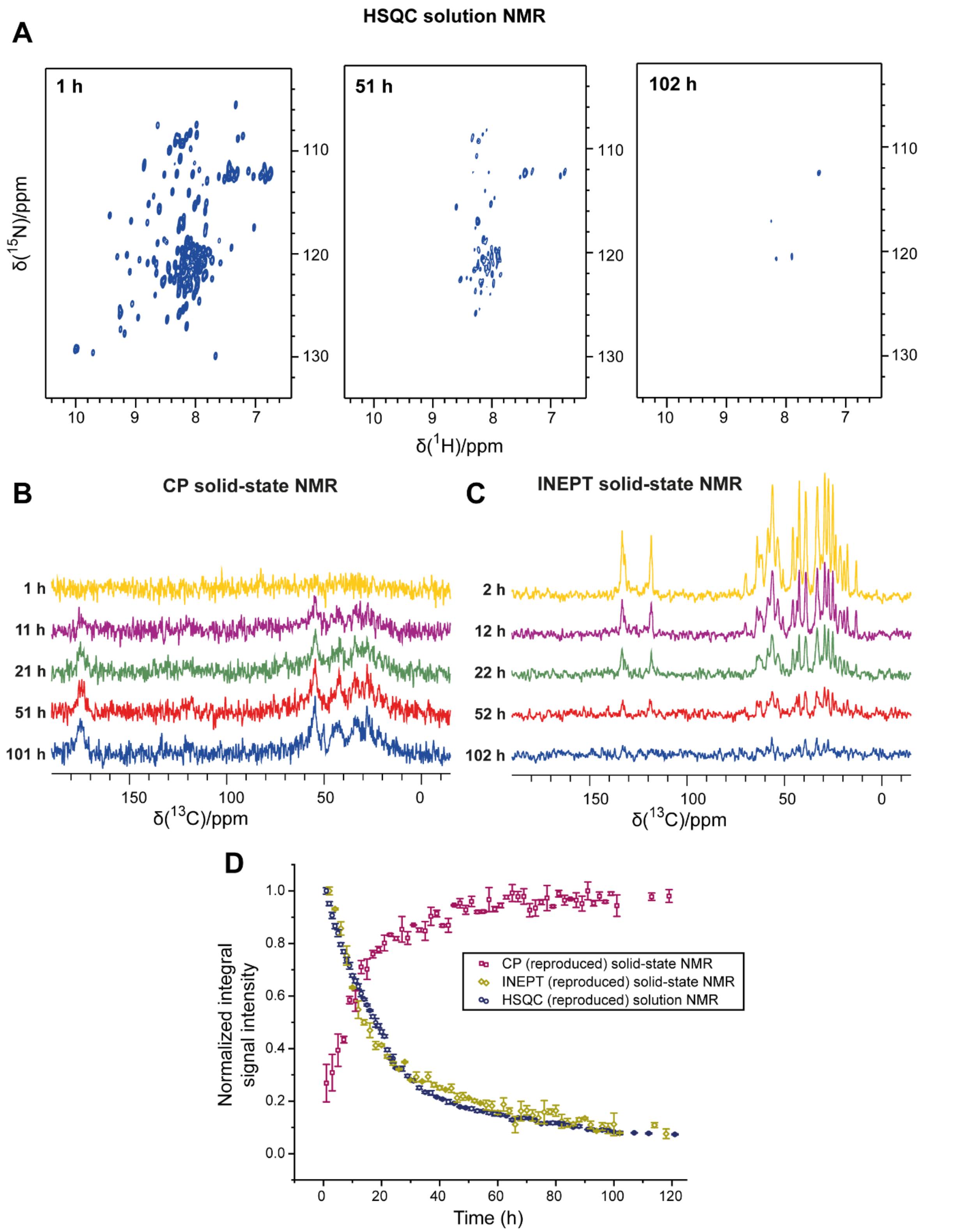

Supplementary Figure S5: NMR spectra of the second (repeated) experiment. The NMR experiments were repeated a second time under identical conditions to assess the reproducibility of the data. NMR spectra and integrated intensities of the repeated (reproduced) aggregation experiments of bovine PI3K-SH3 at 25°C indicated duration after the start of the experiment. A) Representative solution NMR 2D HSQC spectra of the NMR time series, recorded at a magnetic field strength of 14.1 T (600 MHz ^{1}H Larmor frequency) and 25°C. B and C) Representative spectra of ^{13}C-detected 1D solid-state NMR

measurements: CP spectra (B) and INEPT spectra (C). D) Integrated CP, INEPT, and HSQC spectra intensities during the NMR time series.

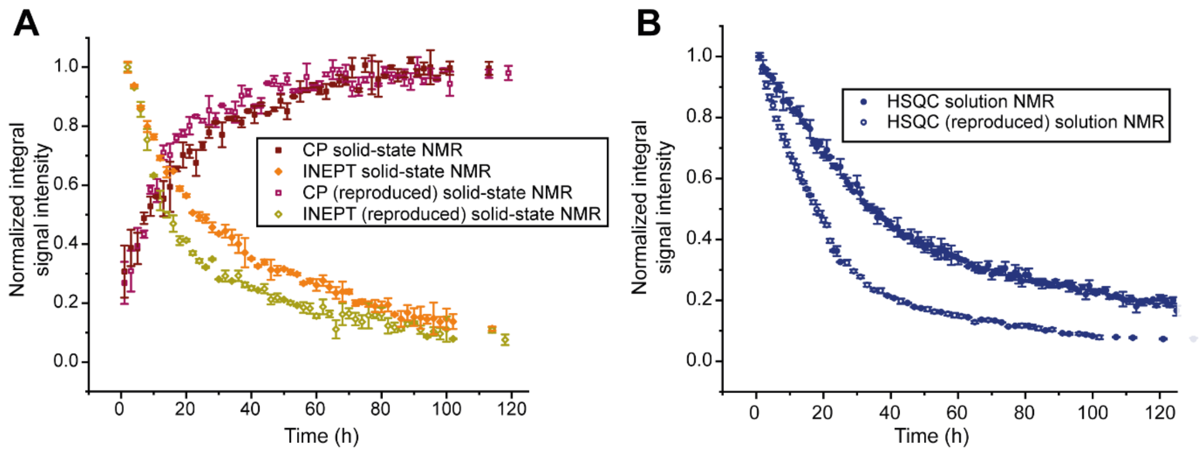

Supplementary Figure S6: Comparison of the integrated intensities of our first NMR measurements and our repeated (reproduced) NMR measurements. A) ^{13}C-detected 1D solid-state NMR experiments, B) First increment of 2D solution NMR ^{1}H-^{15}N HSQC experiments.

Supplementary Table S3: Fitting parameters of decay and build-up curves (mono-exponential fit).

**Spectrum**	τ	$\pm\Delta\tau$
CP solid-state	26.3	1.2
INEPT solid-state	30.2	1.1
INEPT HR-MAS	24.0	0.6
HSQC HR-MAS	25.0	1.2
HSQC solution NMR	37.4	0.6
CP solid-state (reproduced)	14.5	0.7
INEPT solid-state (reproduced)	17.4	0.8
HSQC solution NMR (reproduced)	21.8	0.5

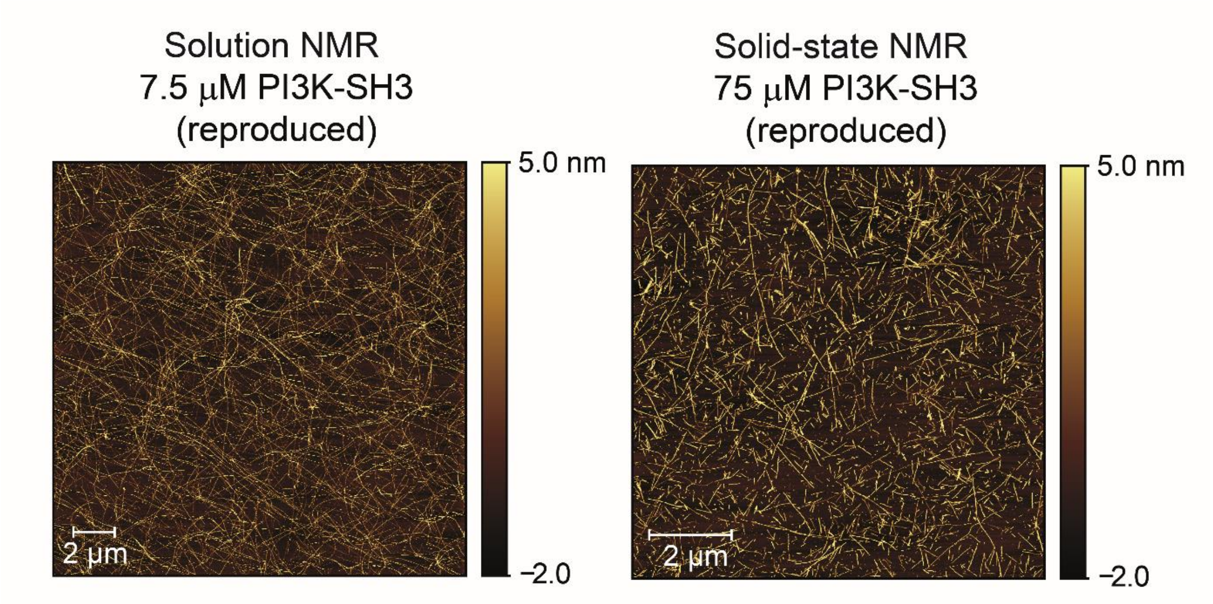

Supplementary Figure S7: AFM images of the repeated (reproduced) measurement. The PI3K-SH3 fibril morphology is highly similar and reproducible to what has been observed before. In the sample obtained after solid-state NMR, in addition to several μm long fibrils, shorter fibrils, likely due to fragmentation or secondary nucleation as an alternative mechanism, are also visible (see Figure 4 for comparison).
